# Effect of Sterilization Methods on Electrospun Scaffolds Produced from Blend of Polyurethane with Gelatin

**DOI:** 10.3390/jfb14020070

**Published:** 2023-01-28

**Authors:** Vera S. Chernonosova, Ilya E. Kuzmin, Inna K. Shundrina, Mikhail V. Korobeynikov, Victor M. Golyshev, Boris P. Chelobanov, Pavel P. Laktionov

**Affiliations:** 1 Institute of Chemical Biology and Fundamental Medicine, Siberian Branch, Russian Academy of Sciences, 630090 Novosibirsk, Russia; 2 Vorozhtsov Novosibirsk Institute of Organic Chemistry, Siberian Branch, Russian Academy of Sciences, 630090 Novosibirsk, Russia; 3 Budker Institute of Nuclear Physics, Siberian Branch, Russian Academy of Sciences, 630090 Novosibirsk, Russia

**Keywords:** electrospun scaffold, sterilization, polyurethane, gelatin, ethylene oxide, electron beam irradiation, protein

## Abstract

Fibrous polyurethane-based scaffolds have proven to be promising materials for the tissue engineering of implanted medical devices. Sterilization of such materials and medical devices is an absolutely essential step toward their medical application. In the presented work, we studied the effects of two sterilization methods (ethylene oxide treatment and electron beam irradiation) on the fibrous scaffolds produced from a polyurethane-gelatin blend. Scaffold structure and properties were studied by scanning electron microscopy (SEM), atomic force microscopy (AFM), infrared spectroscopy (FTIR), a stress-loading test, and a cell viability test with human fibroblasts. Treatment of fibrous polyurethane-based materials with ethylene oxide caused significant changes in their structure (formation of glued-like structures, increase in fiber diameter, and decrease in pore size) and mechanical properties (20% growth of the tensile strength, 30% decline of the maximal elongation). All sterilization procedures did not induce any cytotoxic effects or impede the biocompatibility of scaffolds. The obtained data determined electron beam irradiation to be a recommended sterilization method for electrospun medical devices made from polyurethane-gelatin blends.

## 1. Introduction

In the list of polymers used for the fabrication of scaffolds for tissue regeneration [1,2,3], polyurethanes (PU) demonstrate a number of advantages, such as stiffness and elasticity, bio- and hemocompatibility, biostability, and biodegradation [4,5,6,7]. Medical devices produced from polyurethane are used in medical practice as devices having short- (catheters, tubes, etc.) or long-term (implants, patches, etc.) contact with the internal medium of the organism [8,9,10]. Any medical use of such devices implies their sterility. In general, medical devices are sterilized by dry heat, steam autoclaving, chemical treatment, or irradiation [11,12]. All of the sterilization methods used should not change the shape and internal structure of the devices or the chemical structure of their interacting surfaces with tissues and cells in organisms.

Plastic products usually require more delicate sterilization methods due to their lower melting or gel transition temperatures, oxidation abilities, or the formation of intermolecular bonds that interfere with the mechanical and chemical properties of the devices. For example, it has been previously shown that PU-based scaffolds cannot be sterilized by steam autoclaving (and, of course, dry heat), as this process can lead to degradation and loss of shape [13]. Producers of polyurethanes, such as Lubrizol Inc., do not recommend dry heat treatment or autoclaving of the devices practically produced for all PU, except for Tecobax™, a non-softening, low-tack, highly resilient material that is offered for torque transference [14]. Other PU are recommended to be sterilized with ethylene oxide, peroxide, e-beam or gamma irradiation.

Irradiation is not recommended for aromatic PU due to potential crosslinking of the polymer chains, because sterilization of PU catheters made of Pellethane 2363-80AE by e-beam irradiation leads to a change in the structure of the polymer (branching of polymer chains) [15]. However, this change has practically no effect on the mechanical properties of the materials. Ethylene oxide and peroxide treatment are recommended for devices produced from all PU types, but it is assumed that these products are made by injection molding. Such devices have a low surface-to-weight ratio, and thus the events that randomly occur at their surfaces do not interfere with their mechanical and chemical properties. When looking for fiber materials produced by electrospinning that have a large specific surface, oxidation and irradiation effects may be more visible. The surface topography and biocompatibility in vitro of electrospun Tecoflex SG-80A-based scaffolds have been shown to be changed after ethylene oxide sterilization and UV-ozone sterilization of the materials [16]. In another study, the influence of five sterilization/disinfection techniques (antimicrobial solution, ethanol solution, UV irradiation, gamma irradiation, and e-beam irradiation) was studied for electrospun ChronoFlex C75D materials [17]. It was found that radiation methods and immersion in ethanol led to a significant change in the porosity of the material and its Young’s modulus, as well as a decrease in the viability of cells cultured in contact with the material. Therefore, only acceptable, evidence-based techniques of sterilization should be utilized when treating large surface fiber materials with sterilizing agents, because doing so can affect their properties.

Electrospinning (ES) makes it possible to produce scaffolds from polymers and different blends, including blends of synthetic and natural polymers [18,19,20]. The scaffolds produced from such blends possessed novel properties such as increased stiffness, and hemo- or biocompatibility [21,22]. Mixtures of PU and gelatin have higher bio- and hemocompatibility, as previously demonstrated [23,24,25] and confirmed in an independent study by our Korean colleagues [26]. Thus, materials obtained from PU-gelatin blends are promising scaffolds for tissue engineering. Therefore, the study of the effects of sterilization on such materials prior to their clinical application is an important and urgent task.

In the presented study, we compared the influence of widely used sterilization methods like ethylene oxide treatment and electron beam irradiation on the properties of fibrous scaffolds produced from polyurethane Tecoflex EG-80A with gelatin blends by electrospinning.

## 2. Materials and Methods

### 2.1. Fabrication of Electrospun Scaffolds and Flat Films

The scaffolds were produced using an NF-103 (MESS, Fukuoka, Japan) electrospinning device under the conditions previously described [24]. PU-gelatin scaffolds were made at a collector rotation speed of 300 rpm, a voltage of 18.5–19 kV, and a solution flow rate of 1.1–1.2 mL/h. The electrospinning solution was composed of 3% Tecoflex EG-80A polyurethane (batch 01016300734, Lubrizol Advanced Materials, Beveren, Belgium) (*w*/*v*) and 15% gelatin from porcine skin (Sigma № G2500, Saint Louis, MI, USA) (*w* (gelatin)/*w* (PU)) in 1,1,1,3,3,3-hexafluoroisopropanol (Sigma, Saint Louis, MI, USA). After preparation, scaffolds were dried in a fore vacuum for at least 3 h to remove residual solvent before being stored in sealed zip-lock bags.

Flat films (100–120 µm thick) were obtained by casting a glass slide with 3% Tecoflex EG-80A polyurethane solution (*w*/*v*) in 1,1,1,3,3,3-hexafluoroisopropanol at room temperature (23–25 °C). After 3 days of incubation, fabricated flat films at room temperature were additionally dried using a fore vacuum to remove the residual solvent and stored in sealed zip-lock containers.

### 2.2. Sterilization of PU-Based Scaffolds

#### 2.2.1. Ethylene Oxide Method

Scaffolds and flat films were placed in SteriT^®^ bags (NPO Vinar, Moscow, Russia) and treated using ethylene oxide (EtO) at a concentration of 650 mg/L for 4 h at room temperature with preliminary evacuation and final purge of the sterilization camera (within a validated protocol of Angioline Ltd. (Novosibirsk, Russia)).

#### 2.2.2. Irradiation

Scaffolds and flat films were placed in SteriT^®^ bags (NPO Vinar, Moscow, Russia) and treated with electron beam irradiation using a ILU-6 pulse radio frequency electron accelerator (BINP, Novosibirsk, Russia). The irradiation dose for the samples was 15 kGy (electron beam energy of 2.45 MeV).

### 2.3. Assessment of Sterility of Scaffolds after Various Methods

The efficiency of sterilization methods was studied using a bacteriological method as described in ISO 11737-1:2018 [27]. Scaffold samples without and after sterilization were incubated in a nutrient medium for microorganisms (SOB, Becton Dickinson Inc., Franklin Lakes, NJ, USA) at 30 ± 2 °C for 12 h. The obtained extracts were then analyzed in the Testing Laboratory Center JSC MC “Biotechnopark” (Novosibirsk, Russia) to determine the presence of bacteria or fungi.

### 2.4. Physical and Chemical Properties of Scaffolds

The scaffold structure before and after different sterilizations was examined using a scanning electron microscope EVO 10 (Carl Zeiss AG, Jena, Germany) as earlier described [28]. The fiber diameter and pore size were assessed in SEM images according to ISO 7198:1998 [29]. The *n* = 90–100 fiber diameters and *n* = 80–100 pore sizes were measured using SmartTiff (Zeiss, München, Germany) per each scaffold sample.

Surface roughness was studied using a Multi-Mode 8 atomic force microscope (Bruker, Karlsrue, Germany) with a scanning area of 20 × 20 µm. Images were captured in tapping mode under atmospheric conditions using a NSG10 AFM cantilever (NT-MDT, Moscow, Russia) with a radius of the tip curvature of 6–10 nm. NanoScope Analysis (Bruker, Karlsrue, Germany) and Gwyddion [30] were used to process the AFM images. All of the data presented in this study were generated with cantilevers, for which the spring constant was about 40 N/m and at a scan rate of 0.5–1 Hz.

The contact angle was determined with a Drop Shape Analyzer DSA25 (Kruss GmbH, Hamburg, Germany) using water as a solvent. The drop volume was set to 1 µL and the camera speed was set to the manufacturer’s recommended 160 frames per second.

A Tensor 27 spectrometer (Bruker, Germany) was used to record the FTIR spectra of electrospun scaffolds and flat films (before and after sterilization). The attenuated total reflection (ATR) spectra covered an infrared region of 500–4000 cm^–1^ and were performed on 2–3 different samples. The IR spectra were normalized to the band 2854 cm^−1^.

Mechanical properties were measured using a Zwick/Roell Z10 (Northeim, Germany) universal testing machine with an elongation rate of 10 mm/min as described in ISO 7198:1998 [29]. At least 3–4 samples (50 mm × 10 mm, dog bone shape) per material were tested to determine ultimate tensile strength and ultimate elongation following various methods of sterilization.

For studying the linear dimensions of scaffolds before and after sterilization, 12 mm diameter disks were used and measured using a Vernier caliper, with an accuracy of 0.1 mm.

### 2.5. In Vitro Cell Behavior of Scaffolds after Sterilization

Human fibroblasts were obtained from Dr. A. Cherepanova [31] and used for evaluating cell viability. The study was approved by the ethical committee of ICBFM SB RAS. Cell viability was tested using the Alamar Blue assay (Invitrogen, San Diego, CA, USA) after a 48-h incubation of cells with scaffolds, as previously described [23]. Briefly, cells were seeded on discs from different materials (diameter of 10 mm), which were placed in the wells of a 48-well plate and pressed down with polytetrafluoroethylene o-rings. Cells were incubated in culture medium (DMEM, 10% FBS, 100 U/mL penicillin, and 100 U/mL streptomycin in an atmosphere of 5% CO_2_ at 37 °C) at a dose 1–8 × 10^3^ cells per well on the scaffolds and on the wells without scaffolds (but with a polytetrafluoroethylene o-ring) as a control. After 48-h incubation, the medium was removed, and the cells were incubated in medium without phenol red containing 10% Alamar Blue dye for 6–8 h in a CO_2_-incubator. Obtained supernatants were measured using a Multiskan GO spectrophotometer (ThermoScientific, Waltham, MA, USA) at a 570 nm wavelength and a reference wavelength of 620 nm for the dependence of optical density on the number of planted cells.

Cell adhesion on the scaffold surface was studied by SEM. Samples with fibroblasts were prepared for SEM as follows: after 48-h cultivation, the culture medium was removed from the wells; matrices were washed twice with phosphate buffer, fixed with 2% formaldehyde in physiological saline solution for 30 min, washed thrice with H_2_O, and air-dried. To calculate the mean area of cells on the material, 30 cells from randomly selected SEM images were analyzed.

### 2.6. Statistical Analysis

All data are expressed as the mean ± standard deviation of the mean. Results with *p*-values below the conventional 5% threshold were regarded as significant.

## 3. Results and Discussion

In this study, we used electrospun materials produced from a blend of 3% Tecoflex EG-80A polyurethane with 15% gelatin (Sigma № G2500, Saint Louis, MI, USA). Previously, we have shown that the scaffolds of this composition demonstrate advantages over other blends of PU with gelatin (5, 10, and 20%) in terms of their biocompatibility and mechanical properties in experiments in vitro [24]. Data from the independent study showed that scaffolds obtained from a blend containing 3% Tecoflex EG-80A and 15% gelatin could be recommended as a promising material for the production of cardiovascular devices [25,26].

### 3.1. Characterization of Scaffolds before and after Sterilization

Results of the visual test and sterilization efficiency of scaffolds after treatment with ethylene oxide and electron beam irradiation are summarized in Table 1. In the scaffold sterilized with ethylene oxide, shrinkage and color changes were observed. It was found that the change in material dimensions was committed to 25 ± 1% with a proportional increase in their thickness. The color change of PU-based materials after sterilization by γ-irradiation has been previously detected in other works [32,33]. Actually, the sterilization dose is usually determined based on the admissible biological burden on the final product. The absorbed dose of 1.8–2 kGy results in a tenfold decrease in bioburden [33] for the majority of microbes. The 25 kGy dose is assumed to be a default sterilization dose for medical products. However, the practical sterilization dose range is 15–25 kGy, depending on the initial bioburden, required sterility level, and radiation degradation of the treated products. An analysis of sterilization effectivity showed that both used methods led to the destruction of microorganisms (Table 1), confirming 15 kGy as a sterilization dose for electrospun materials.

**Figure 1:**
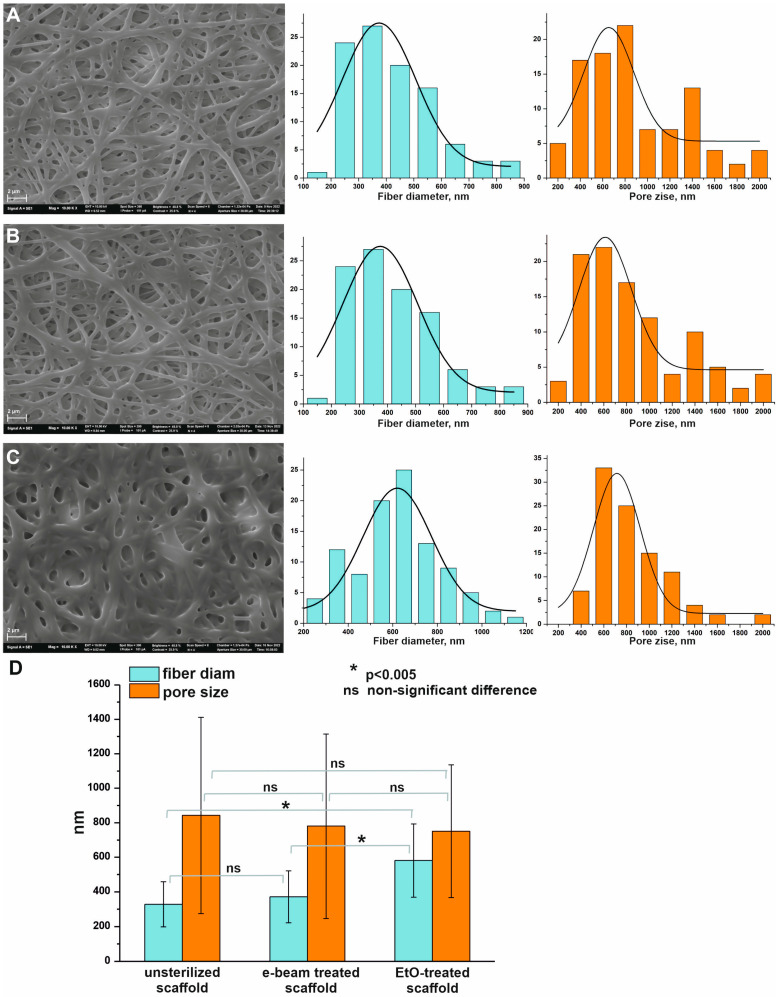
SEM images of scaffolds obtained from a PU-gelatin blend before and after sterilization and distribution of structural parameters (fiber diameter—cyan color diagrams, pore size -orange color diagrams). (**A**)—unsterilized scaffold; (**B**)—scaffold sterilized with electron beam irradiation; (**C**)—scaffold sterilized with ethylene oxide; (**D**)—statistical analysis of the data.

The SEM images of the scaffold before and after sterilization are presented in Figure 1. Data demonstrates different evaluations of surface morphology in response to the sterilization method. Figure 1C shows that the formation of glued-like structures at the junctions of the fibers and the change in fiber diameter were only specific for scaffolds treated with ethylene oxide. The fiber diameter in the scaffolds does not change following the exposure to irradiation (371 ± 149 nm), but increases after treatment with EtO (581 ± 212 nm). It has been shown that a scaffold exposed to EtO reduces both the number and size of pores in the material compared to a non-sterile scaffold and a scaffold sterilized with electron beam irradiation (Figure 1D). The obtained data are consistent with the data previously described [17]. It was shown that the treatment of electrospun materials made from polyurethane ChronoFlex C75D with ethylene oxide led to an increase in the diameter of the fibers and a decrease in the porosity of the material. Increasing fiber diameters and decreasing pore sizes are obviously concerned with the shrinkage of scaffolds after EtO treatment.

**Figure 2:**
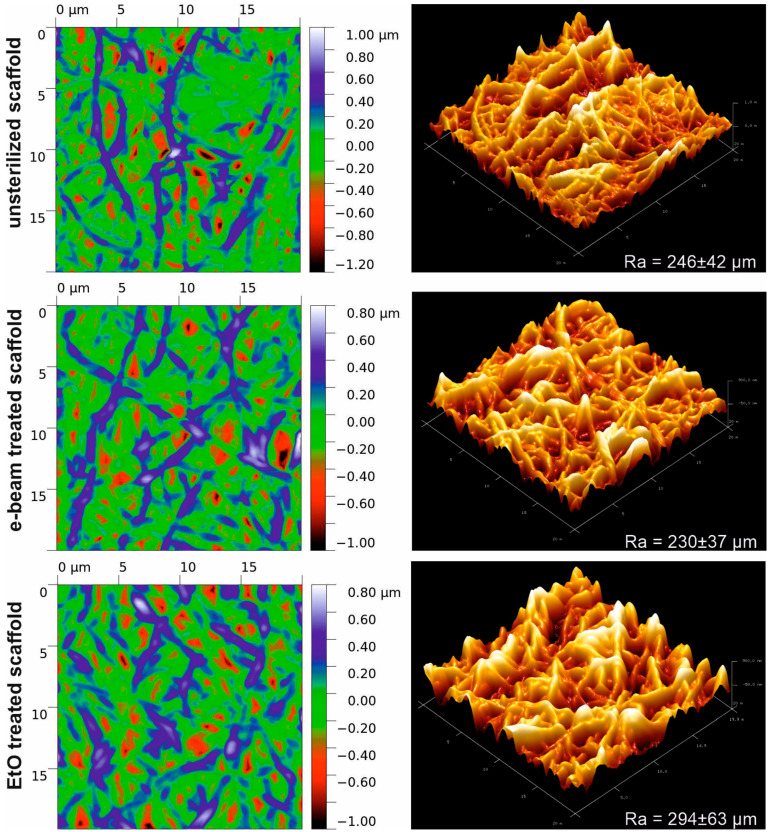
AFM images of the surface of electrospun scaffolds obtained from a PU-gelatin blend before and after sterilization.

AFM dates (Figure 2) demonstrate a notable increase in the surface roughness of scaffolds after EtO treatment (Ra = 294 ± 63 µm) as compared to untreated or electron beam irradiated samples (Ra = 246 ± 42 µm and 230 ± 37 µm, respectively). At those maximal ranges of peak-hole height difference, the studied scaffolds are very similar, but the EtO-treated scaffold is characterized by fewer sharp peaks.

### 3.2. Effects of Sterilization Methods on Mechanical Properties of Electrospun Scaffolds

**Figure 3:**
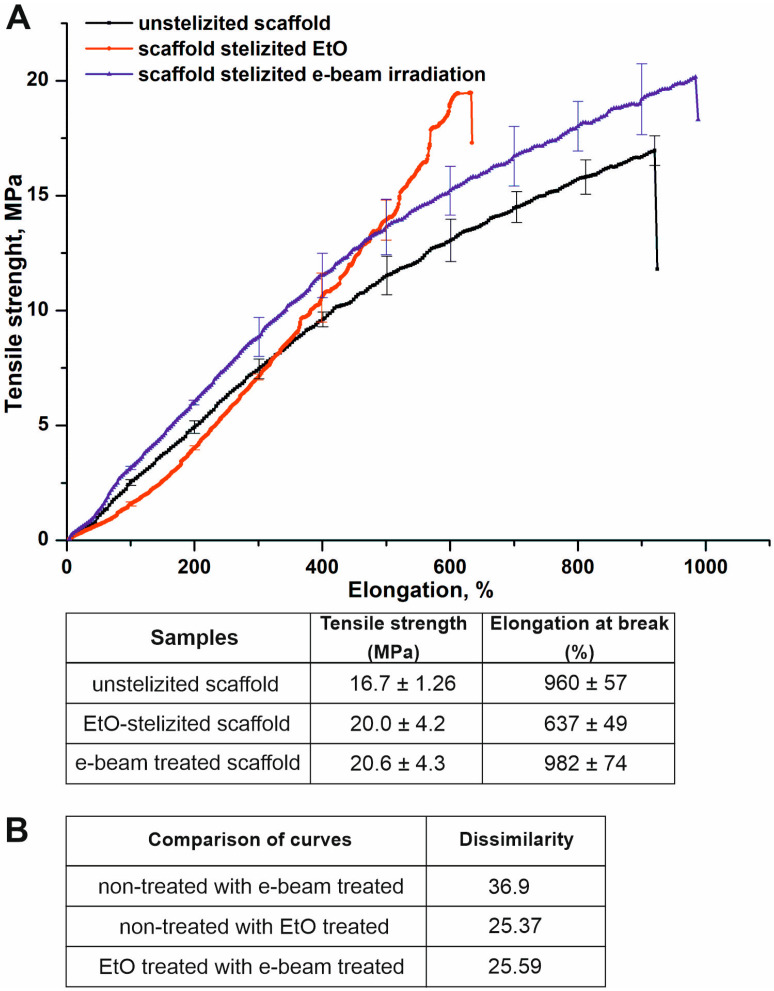
Influence of sterilization on mechanical properties of the scaffolds. (**A**)—typical tensile stress diagrams of scaffolds before and after sterilization. The plots are presented as the mean of the scaffold strength measured in three samples, bars represent the standard error. (**B**)—difference in curves for the tensile stress diagrams of materials was obtained using Kolmogorov-Smirnov test.

It is known that changes in the structure of materials affect their mechanical properties [34,35]. The mechanical properties of scaffolds before and after sterilization were compared. The typical stress-strain plots for tested samples are shown in Figure 3. Sterilization by both methods induced principal changes in the mechanical properties of the materials, as confirmed by profile assays using the Kolmogorov-Smirnov test [36]. The values of the difference of curves for the tensile stress diagrams of the materials after sterilization vary in the range from 25.37 to 36.9 (Figure 3B). The maximum value is detected by comparing the unsterilized scaffold with the electron irradiated scaffold. However, sterilization methods have been found to affect the tensile strength and elongation of materials. Treatment by EtO and e-beam irradiation correspondingly increased the tensile strength of electrospun scaffolds up to 20.0 ± 4.2 ÷ 20.6 ± 4.3 MPa compared with an unsterilized sample demonstrating 16.7 ± 1.26 MPa tensile strength. Scaffolds after treatment by EtO were characterized by about 30% less ultimate elongation (637 ± 49%) compared to control scaffolds (960 ± 57%) and scaffolds sterilized by electron beam irradiation (982 ± 74%).

The obtained data indicate an increase in fiber diameter, the formation of glue-like structures, and a decrease in two-dimensional linear sizes in materials treated with EtO. Obviously, these facts are responsible for the increase in ultimate strength and decrease in ultimate elongation of EtO-treated scaffolds. The materials with more inter-fiber junctions and thicker fibers were found to be stronger and less elastic [34,37]. In contrast, irradiation did not drastically change the structure of the scaffolds, as was shown by SEM. Actually, ionized radiation energy is absorbed in matter and first excites its atoms and molecules, creating secondary electrons, ions, photons, and free radicals [38] that react with other atoms and molecules. Finally, thermalization processes convert input energy into heat, which can then be measured and used to calculate the absorbed dose. The absorbed dose of 1 Gy is determined as 1 Joule per 1 kg of matter [38]. Therefore, a 1 kG dose equals one joule per gram, 0.24 calories per gram, or a 0.24 °C increase in water temperature. Other materials are heated in inverse proportion to their heat capacities. Given that ΔT for most plastics ranges between 0.4 and 0.7 °C/kGy, a 15 kGy dose will heat plastics to 6.5–11 °C. The melting point of Tecoflex EG-80A is higher than 200 °C and the thermo-irradiation effect is evidently not able to induce inter-fiber crosslinks. Apparently, irradiation with an electron beam leads to the cross-linking of polymer molecules, which increases the tensile strength of scaffolds [39].

### 3.3. Effects of Sterilization Methods on Chemical Properties of Electrospun Scaffolds

**Figure 4:**
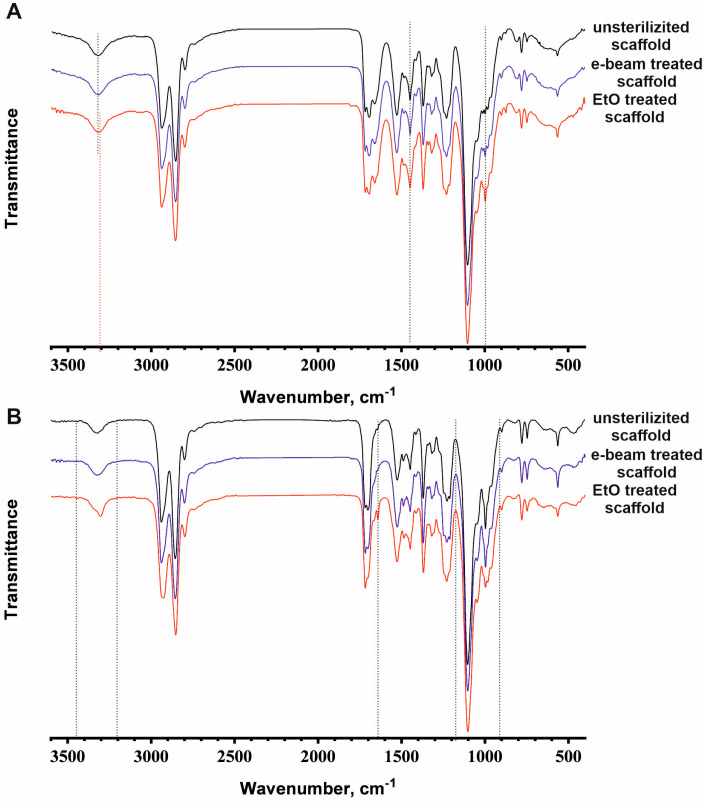
ATR spectra data or FTIR analysis of the electrospun scaffolds from the PU-gelatin blend (**A**) and flat films from PU (**B**) before and after sterilization. Changes in the spectra are marked by vertical dotted lines.

To characterize the materials’ surface after sterilization, we used the FTIR analysis (Figure 4) and the measurement of the contact angle (Table 2).

The ATR spectrum of the scaffold sterilized using electron beam irradiation does not differ in shape and size of individual peaks or the splitting of peaks compared with the spectrum of the unsterilized scaffold (Figure 4A). Changes in ATR spectra were detected between the unsterilized scaffold and the EtO-sterilized scaffold in the peak shift of the absorption bands from 3324 to 3307 cm^−1^ (stretching vibrations of NH groups) and in the increase in band intensity at 1435 cm^−1^ (bending vibrations of CH_2_ groups in –CH_2_-CO-) and 997 cm^−1^ (stretching vibrations of C-O-C). Such changes in PU-gelatin scaffolds indicate the occurrence of chemical reactions in the polymers, which must be taken into account. Under sterilization conditions, EtO can easily react with various types of amino groups in proteins [40,41]. Secondary amino groups in the structure of urethane fragments (NH-C(=O)-O) can interact with ethylene oxide in a similar way to primary amino groups, but at a slower rate. The ATR spectrum demonstrates modification of protein-free PU flat films after EtO sterilization in the following regions: 3360–3180 cm^−1^ (stretching vibrations of NH groups), 1630 cm^−1^ (amide I), and 1150–900 cm^−1^ (stretching vibrations of C-O-C) (Figure 4B). Thus, in addition to protein modification by EtO in electrospun scaffolds, it can also react with PU. Considering the nanoporous structure of fibers as a result of solvent evaporation, the outer surface and inner space of the fibers are easily accessible for EtO modification. The extensive modification of the PU-gelatin scaffolds leads to charge redistribution and can change the fiber structure observed using SEM and AFM, resulting in an alteration of the material’s stiffness and elasticity.

Actually, treatment of the scaffold with ethylene oxide leads to a significant increase in its hydrophobicity (contact angle = 122.95 ± 1.58°, *p* < 0.05) compared to other types of scaffolds (contact angle 114.63 ± 1.03 ÷ 117.90 ± 1.74°). Similar data were demonstrated for materials made from a polyurethane/polylactide blend [42,43]. The authors observed an increase in contact angle after EtO treatment and linked it to the opening of the EtO ring as a strongly reactive molecule, followed by a radical reaction modification of the polymer chain ends. Exposure of the proteins on the surface of scaffolds produced from blends of Tecoflex EG-80A and gelatin provides even better conditions for such modification [24].

It could be expected that the attachment of polar ethylene oxide groups to the matrix surface during ethylene oxide sterilization would lead to a decrease in the contact angle. It has been shown that modification of Tecoflex EG-80A polyurethane by grafting polyethylene glycol to urethane fragments of macromolecules increases hydrophilicity, and reduces the contact angle of water contact with polymer films due to an increase in the number of polar hydroxyl groups and the ether linkages [44]. Nonetheless, EtO modification that results in N-2-hydroxyethyl groups increases hydrophobicity.

It should be noted that the contact angle of the initial scaffolds equal to 114.63 ± 1.03° is significantly higher than the contact angles separately measured in other works for Tecoflex EG-80A polyurethane films and gelatin. Contact angles were estimated to be 71 ± 1° for Tecoflex EG-80A polyurethane films [44] and 70–79° for various gelatin films [45].

A contact angle of 114° is typical for hydrophobic polymer films such as Teflon. The high value of the contact angle for the tested scaffold as compared with polyurethane and gelatin films is associated with a number of factors—a change in the orientation of the functional groups of polymers in the process of drawing the fibers, the interaction of the functional groups of gelatin and polyurethane, and the morphology of the matrix (pore diameter, fiber thickness, surface roughness). It is known that the surface roughness [46] of polymeric materials can affect the contact angle to a greater extent than a change in the chemical composition of the material. An increase in the contact angle of scaffolds after sterilization by irradiation and ethylene oxide is primarily associated with a change in the morphology of the matrices (changes in fiber thickness, pore size, etc.). In contrast to irradiation, EtO treatment can penetrate into fibers and lead to the modification of outer and inner fiber spaces, leading to strong shrinkage and changes in the morphology of scaffolds (Figure 1) and, obviously, fibers. Changes in the chemical structure of the fiber surface after EtO treatment, as confirmed by ATR spectra, as well as their physical structure and scaffold roughness, can all contribute to an increase in contact angle.

### 3.4. Effects of Sterilization Methods on Interaction of Cells with Scaffolds

**Figure 5:**
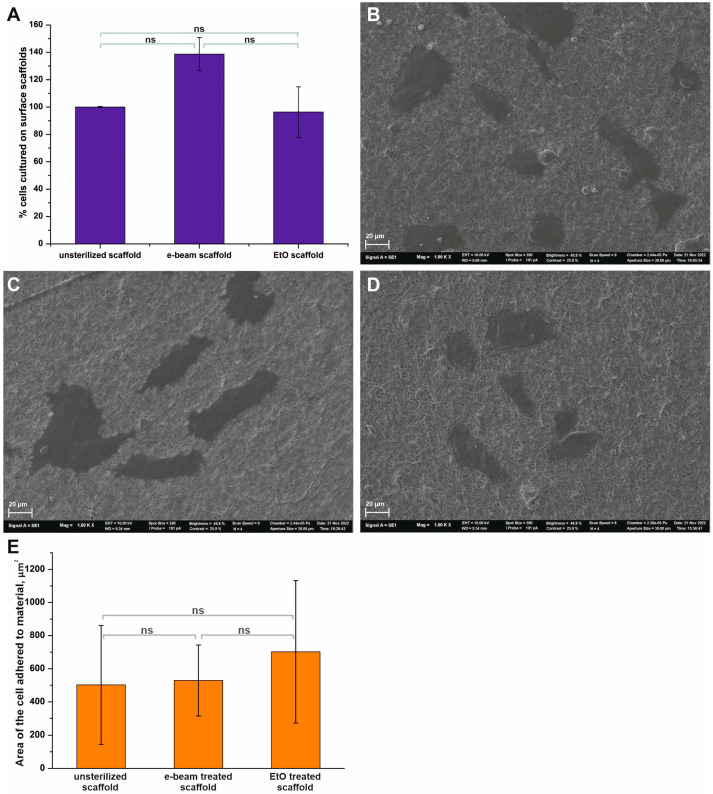
The interaction of fibroblasts with the surfaces of different scaffolds. (**A**)—Cell viability was tested by Alamar Blue assay after 48-h incubation of cells with scaffolds (Mean ± S.D. of three experiments). (**B**)—SEM image of cells on the surface of an unsterilized scaffold. (**C**)—SEM image of cells on the surface of a scaffold sterilized using e-beam irradiation. (**D**)—SEM image of cells on the surface of a scaffold sterilized using ethylene oxide. SEM images have a magnification of 1000. (**E**)—Area of cell adhered to the surface materials. The value bars with ns are not significant.

Considering the change in the surface structure and chemical composition of matrices, we evaluated the interaction of primary human fibroblasts with materials before and after sterilization. The assessment of cells’ interaction with the surface of materials was examined after 48 h of seeding using a test for cell viability and SEM (Figure 5).

The results of the Alamar Blue test demonstrate that sterilization methods have an effect on interactions with fibroblasts (Figure 5A), but do not induce any cytotoxic effects. The highest viability (138.7 ± 12.1%) was observed for human fibroblasts grown on scaffolds sterilized using electron beam irradiation. In comparison, treatment of the scaffold with ethylene oxide slightly altered cell viability (96.3 ± 18.5%) compared to the control sample. However, the results of cell viability depending on the type of material did not significantly differ; the *p*-value was equal to 0.0868. The SEM data showed that the fibroblasts had a proper morphology, with a flattened cell body for the studied scaffolds (Figure 5B–D). The average cell area on the surface of the materials was calculated using the SEM data (Figure 5E). It was found that the sterilization of scaffolds did not significantly increase the area, which was occupied by cells on the surface.

Cell adhesion is known to be related to surface roughness [47,48] and surface chemical structure [49]. The highest surface roughness value was found for EtO-treated scaffolds, as demonstrated by AFM. At the same time, non-treated and irradiated scaffolds had sharp peaks as compared to EtO-treated material. Other researchers have found that a change in the surface roughness of materials in a range of 0.67–4.7 μm led to an increase in adhesion and proliferation of cells [47,48]. The absence of effects in our case is possibly for the following reasons. Firstly, the change in the surface roughness of the studied materials is very minimal, which does not affect cell adhesion on their surface. Secondly, as previously demonstrated, the surface layer of scaffolds produced from a blend of synthetic polymers with proteins readily exposes proteins at their surface fibers [28]. The data obtained demonstrate that fibroblasts adhere and proliferate at the surface of scaffolds in a way that is no worse than at the surface of tissue culture plastic. As a result, all changes caused by the treatment of the scaffold with EtO and observed using SEM, AFM, and FTIR did not critically affect cell adhesion and proliferation.

## 4. Conclusions

Considering that medical electrospun devices made from PU-protein mixtures can be recommended for clinical practice, the choice of a method for their sterilization is a significant step towards their use. The effect of sterilization methods on the properties and characteristics of materials made from PU blended with gelatin has never been described before. The data obtained in this study demonstrate that ethylene oxide treatment interferes with all listed scaffold properties (linear dimensions, structure parameters, mechanical properties), and cannot be recommended despite the fact that it does not interfere with scaffold biocompatibility. Electron beam irradiation increases the tensile strength of scaffolds and does not interfere with other tested properties. Based on the obtained data, it is possible to recommend electron beam irradiation as a sterilization protocol for electrospun materials produced from PU-gelatin blends.

## Figures and Tables

**Table 1:** The change in visual parameters and the sterilization efficiency.

Parameter	Unsterilized Scaffold	e-Beam Treated Scaffold	EtO Treated Scaffold
Shrinkage	N	N	25 ± 1
Color	N	N	slightly yellow
Microorganisms	gram(+) spore- forming bacillus	absence of bacteria and fungus	absence of bacteria and fungus

**Table 2:** Effect of the EtO and e-beam treatment on the hydrophobicity of the surface of the scaffolds.

Material	Contact Angle	Different (*p* < 0.05)
unsterilized scaffold	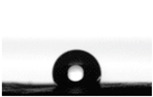	EtO-treated scaffold
	114.63 ± 1.03°	
e-beam-treated scaffold	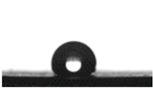	EtO-treated scaffold
	117.90 ± 1.74°	
EtO-treated scaffold	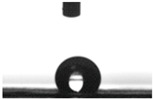	unsterilized scaffold; e-beam-treated scaffold
	122.95 ± 1.58°	

## Data Availability

Data presented in this study are available on request, owing to privacy and ethical restrictions.
